# The combination of anti-PD-1 antibodies, trastuzumab and chemotherapy may improve the outcome of some patients with HER2-positive alpha-fetoprotein-producing gastric cancer: a retrospective real-world analysis from a single center

**DOI:** 10.1186/s12885-025-14808-3

**Published:** 2025-10-10

**Authors:** Shaohua Ge, Feixue Wang, Hongli Li, Yuchong Yang, Le Zhang, Jingjing Duan, Ming Bai, Rui Liu, Tao Ning, Xia Wang, Zhi Ji, Yansha Sun, Ting Deng

**Affiliations:** Department of GI Medical Oncology, Tianjin’s Clinical Research Center for Cancer, Tianjin Key Laboratory of Digestive Cancer, Tianjin Medical University Cancer Institute & Hospital, National Clinical Research Center for Cancer, Tianjin, 300060 China

**Keywords:** Alpha-fetoprotein-producing gastric cancer, HER2-positive gastric cancer, Anti-PD-1 antibody, Objective response rate, Immune microenvironment

## Abstract

**Background:**

Patients with alpha-fetoprotein-producing gastric cancer (AFPGC) characterized with elevated serum AFP levels have received increased interest because of their aggressive behavior and poor prognosis. AFPGC patients who are HER2 positive are even poorer.

**Methods:**

A single-center retrospective real-world analysis was conducted in patients with histologically confirmed gastric cancer, an AFP level > 7 ng/ml and who were pathologically HER2 positive at Tianjin Medical University Cancer Institute. Clinical characteristics, treatments and survival were recorded. The last follow-up date was February 10, 2023. The immune microenvironment was detected via multiple immunofluorescence stains.

**Results:**

From May 2017 to May 2022, 29 advanced patients who were HER2 positive were included in the final analysis of 259 AFPGC patients. The median AFP level was 6745.13 ng/ml. Sixteen patients (61.5%) received the quadruplet combination of doublet chemotherapy, trastuzumab and anti-PD-1 antibody. The ORR and DCR were 66.7% and 91.7% in all patients, and 80% and 93.3% in patients in the quadruple combination group. The median progression-free survival (mPFS) and overall survival (mOS) for all patients were 10.27 and 20.50 months, respectively. For the quadruplet combination group, the mPFS and mOS were 7.47 and 14.87 months, respectively, but there were still 6 patients without disease progression and 8 patients who were alive (PFS and OS range: 7.3–41.07 months). Multiple immunofluorescence stains revealed significantly fewer CD3 + , CD8 + , CD56 + , and CD68 + immune cells in the tumor parenchyma and stroma in HER2-positive AFPGC patients than in HER2-negative and AFP-normal gastric cancer patients.

**Conclusions:**

Compared with current treatments, parts of HER2-positive AFPGC can achieve equivalent ORR and survival benefits, especially with HER2-targeted, anti-PD-1 antibody and chemotherapy.

**Supplementary Information:**

The online version contains supplementary material available at 10.1186/s12885-025-14808-3.

## Introduction

Gastric cancer is one of the most frequently cancer in worldwide and in China with poor prognosis. A comprehensive nationwide cohort analysis of 220,304 gastric cancer patients showed that at least 26.16% of the patients were in stage IV [1]. The prognosis of some special subtype gastric cancer was even poor [2–4], such as Alpha-fetoprotein (AFP)-producing gastric cancer (AFPGC), signet-ring cell carcinoma and so on. AFPGC, also called AFP-positive or AFP-elevated gastric cancer, has gained much attention in recent decades because increasing evidence supports the poor prognosis associated with high serum AFP levels [5–9]. To date, no consensus has been reached on the exact definition or cutoff of AFP positivity, and proportions ranging from 2.9% to 19.1% have been reported in different studies. According to published studies, poor prognosis is related to high cell proliferation, high VEGF expression, high invasion and migration, immune suppression, etc. [7, 10]. The mechanism of AFP immunosuppression is not clear now. It was reported that AFP mRNA expression was positive corelated to the expression of antigen presentation-related molecules by sequencing and TIMER database analysis [3].With next-generation sequencing (NGS), single-cell RNA sequencing and copy number alteration analysis, many markers and pathways, such as *P53*, HGF/MET, *CCNE1, and ERBB2*, are involved in molecular biology [6, 7, 11–13]. Among these markers, ERBB2 positivity is obviously greater than that in normal gastric cancer [14, 15]. Patients with *ERBB2* amplification also have worse survival than those without amplification [6].

The receptor tyrosine-protein kinase erbB-2, also known as HER2, is a member of the EGFR family of receptor tyrosine kinases. In the phase III ToGA trial [16], significantly improved overall survival (OS) and objective response rates (ORRs) have made HER2 a promising therapeutic target, with a median OS of more than 12 months in advanced gastric or gastroesophageal junction cancer patients. Since 2010, trastuzumab (an anti-HER2 antibody) plus conventional chemotherapy has become the standard-of-care treatment for HER2-positive stomach cancer. Then there are more studies on HER-2 examination and anti-HER-2 therapy [17, 18], even on neoadjuvant/conversion therapy of gastric cancer [19]. Based on the categories in breast cancer of low and ultra-low HER-2 expression [20], the categories of HER-2 in gastric cancer is changing and the expression also changed after anti-HER-2 therapy [21]. In addition, another phase III clinical trial, KEYNOTE-811 [22], which added immunotherapy to chemotherapy and anti-HER2 targeted therapy, also showed encouraging results, with an ORR of 74.4%, a disease control rate (DCR) of 96.2% and a complete response rate (CR) of 11.3%. The KEYNOTE-811 study revealed promising opportunities for a stronger combination treatment pattern in HER2-positive gastric cancer patients. Of course, the expression of PD-1/PD-L1 was also related to the effect and side effects of immunotherapy [23–26]. The positive rate of HER-2 in China was 11.47% [1]. Masakazu Fujimoto et al. [27] evaluated the HER2 expression status in two histological subtypes of AFPGC, hepatoid adenocarcinoma and gastric carcinoma with enteroblastic differentiation, and found that HER2 is frequently overexpressed compared with other non-AFPGC subtypes. Given that AFPGC is recognized as a distinct subtype of GC with poor behavior and prognosis, we wondered whether HER2 overexpression or amplification in AFPGC could result in equivalent benefits from current treatments, especially with anti-PD-1 antibody and HER2-targeted therapy.

In this study, we focused on HER2-positive AFPGC. A single-center retrospective analysis was conducted to investigate the clinicopathological characteristics and outcomes of HER2 positive AFPGC patients at Tianjin Medical University Cancer Institute and Hospital. To date, this is the first study with the largest number of HER-2-positive AFPGC patients.

## Patients and methods

### Patient collection

This was a single-center, retrospective study to evaluate the clinicopathological features and outcomes of AFP-producing HER2-positive gastric cancer patients. The study was approved by the independent ethics committees of Tianjin Medical University Cancer Institute and Hospital. All enrolled patients provided written informed consent. Patients were carefully selected according to the following inclusion and exclusion criteria. The inclusion criteria were as follows: 1) pathologically diagnosed primary gastric cancer by endoscopic examination; 2) elevated AFP levels were determined before treatment and after two or three cycles treatment, the cutoff value for the serum AFP level used in this study was 7 ng/ml (the upper limit of normal value from the standard report of the Department of Clinical Laboratory); 3) HER2-positive status (defined as immunohistochemical staining for HER2 3 + or immunohistochemical staining for HER2 2 +/ISH +) confirmed as positive by immunohistochemistry (IHC) and in situ hybridization (ISH); 4) unresectable locally advanced or metastatic disease by CT or PETCT image; 5) available information on first-line therapy and follow-up; and 6) all patients signed informed consent to participate in this study. Exclusion criteria were as follows: chronic hepatitis, cirrhosis, pregnancy, genital tumors and other diseases that might cause elevated AFP. Survival data were still being collected, and the last follow-up date was 10 February 2023.

### Baseline, treatments and follow-up

For patients enrolled in this study, baseline clinicopathological parameters, including age, sex, primary tumor location, pathological type, differentiated grade, metastatic site, AFP level at diagnosis, and HER2 status, along with biochemical parameters, including other tumor markers and D-dimer and albumin (ALB) levels, were collected from the hospital system. Treatments included surgery, chemotherapy, targeted therapy, and immunotherapy. The response evaluation was performed according to RECIST 1.1 according to CT imaging before and during the treatments. Timely follow-up was performed, and treatment information, including first-line drugs, response assessments and survival data, was collected. The last follow-up date was February 10, 2023.

### Multiple immunofluorescence stains to detect the immune microenvironment

Formaldehyde-fixed, paraffin-embedded (FFPE) samples were prepared for detection of the immune microenvironment via multiplex immunofluorescence. The samples were processed via a PANO 7-plex immunohistochemistry kit (Baiano Panorama, Beijing, China), stained with different primary antibodies, incubated with enzyme-labeled secondary antibodies, and amplified with amide signals via a TSA fluorescence kit (Baiano Panorama, Beijing, China). Finally, the nuclei were labeled with 4',6'-diamidino-2-phenylindole (DAPI, cat. D9542, Sigma‒Aldrich). Two combinations of primary antibodies were used: Panel A included Pan-CK antibodies (C11, CST4545, CST), CD3 antibodies (SP7, Ab16669, Abcam), CD8 antibodies (C8/144B, CST70306, CST), PD-1 antibodies (EH33, CST43248, CST) and PD-L1 antibodies (E1L3N, CST13684, CST); Panel B included Pan-CK antibodies (C11, CST4545, CST), CD56 antibodies (123C3, CST3576, CST), CD68 antibodies (BP6036, BX50031, Biolynx), and CD163 antibodies (D6U1J, CST93498, CST).

Stained sections were scanned via a Mantra system (PerkinElmer, Waltham, Massachusetts, US) with the fluorescence broad spectrum set at 420–720 nm and a wavelength interval of 20 nm. Images were analyzed and quantified with inForm software (PerkinElmer, Waltham, Massachusetts, US) to obtain the density and positivity rate of cells labeled with CD3 + (T cells), CD8 + (cytotoxic T cells), CD56 + (NK cells), CD68 + CD163- (M1 macrophages), CD68 + CD163 + (M2 macrophages), PD1 + and PD-L1 + in the tumor parenchyma and stroma.

### Statistical analysis

All data analyses were performed via SPSS 22.0 (SPSS Inc., Chicago, IL, USA). Graphs were generated via GraphPad Prism v.9.0.0 (La Jolla, CA, USA). The chi-square test was used to compare the clinicopathological factors between different subgroups. Cumulative survival was calculated via the Kaplan–Meier method. The log-rank test was used for univariate survival analysis, and the Cox regression model was used for multivariate survival analysis. A two-tailed P < 0.05 was considered statistically significant.

## Results

### Clinicopathological characteristics of HER2-positive AFPGC

**Fig. 1:**
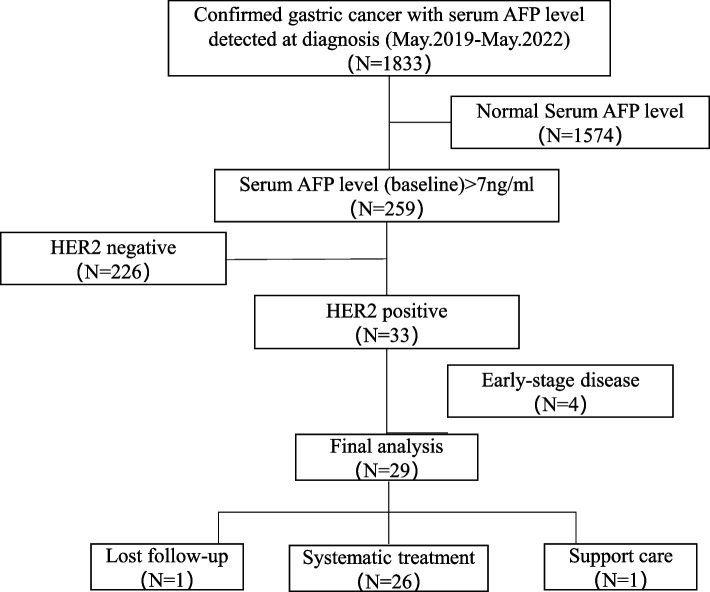
The overall recruiting protocol

From May 2019 to May 2022, 1833 patients with histologically confirmed gastric cancer and serum AFP levels were screened at the Tianjin Medical University Cancer Institute and Hospital, and a total of 259 patients (14.1%) presented with elevated serum AFP levels above the upper limit of 7 ng/ml. Using both immunohistochemistry (IHC) and in situ hybridization (ISH), 33 patients (12.8%) were confirmed to be HER2 positive. Four patients with early-stage disease were excluded, and 29 patients were included in the final analysis. The overall recruitment protocol was shown in Fig. 1.

**Fig. 2:**
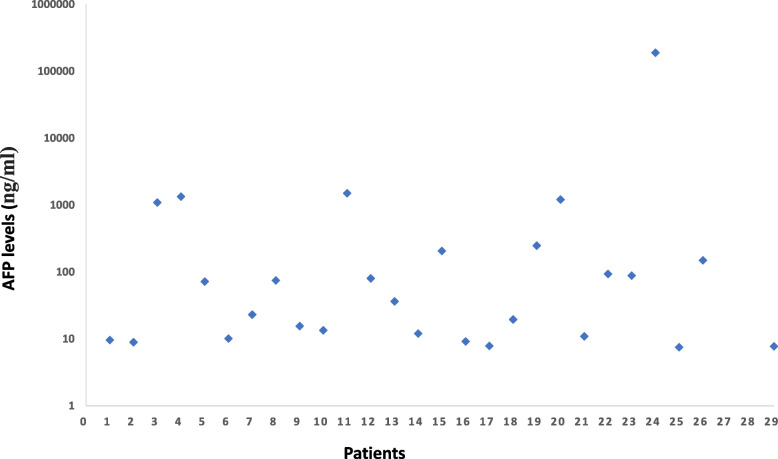
Details of AFP (the AFP levels are shown logarithmically) in each patient

The clinicopathological features summarized in Table 1 revealed that the median age was 60 years (range: 39–90 years), and most patients were male (72.4%). A total of 72.4% of the patients (21 patients) had more than two metastatic sites at initial diagnosis. Lymph node metastasis was observed in 28 patients (96.6%) at initial diagnosis. In addition, more than half of the patients (17 patients, 58.6%) had liver metastasis, and 10 patients (34.5%) had peritoneal metastasis. The median AFP level was 6745.13 ng/ml, ranging from 7.49 to 189,243 ng/ml, with almost half of the patients (14 patients, 48.3%) falling within the interval between 10 ng/ml and 100 ng/ml. High AFP levels (greater than 1000 ng/ml) were observed in 5 patients (17.2%). Apart from one patient with an extremely high AFP level of 189,243 ng/ml, the AFP levels of the other patients were not higher than 6000 ng/ml (the details of the AFP level were shown in Fig. 2). For HER2 status, 79.3% were HER2 3 + and 20.7% were HER2 2 +/ISH +. Among these patients, IHC results for mismatch repair (MMR) proteins were available for 24 patients, and all the patients were proficient in MMR (pMMR).

**Table 1:** Clinicopathological features of HER2-positive AFPGC

Characteristics	Number	%
Age (years)		
Median (range)	60 (38–90)	
Gender		
Male	21	72.4
Female	8	27.6
Smoking		
Yes	13	44.8
No	16	55.2
Drinking		
Yes	15	51.7
No	14	48.3
Pathologic differentiation		
Poor	8	27.6
Moderate	2	17.2
Poor-Moderate	6	20.7
ECOG PS		
0	1	3.4
1	28	96.6
HER2 expression		
3 +	23	79.3
2 +/FISH +	6	20.7
MMR status		
pMMR	24	82.8
dMMR	0	0
unknown	5	17.2
AFP level (ng/ml)		
≥ 1000	5	17.2
100–1000	3	10.3
10–100	14	48.3
≤ 10	7	24.1
Number of metastatic organs at diagnosis		
1	8	27.6
2	15	51.7
≥ 3	6	20.7
Metastatic sites		
Liver metastasis	17	58.6
Lymph node metastasis	28	96.6
Peritoneal metastasis	10	34.5

### Therapeutic details and response evaluation

One patient was excluded from the treatment and prognosis analysis because it was lost to follow-up. Among the remaining 28 patients, two patients with poor performance status, one receiving supportive care and one receiving palliative surgery, died within one month (0.97 months and 0.90 months). A total of 26 patients received systematic anticancer treatment, as summarized in Table 2. The quadruple combination of chemotherapy, trastuzumab, and anti-PD-1 antibodies was the main therapeutic regimen administered to more than half of the patients (16 patients, 61.5%). In addition, four patients (15.4%) received chemotherapy combined with trastuzumab, three patients (11.5%) received chemotherapy and anti-PD-1 antibodies, two patients (7.7%) received chemotherapy alone, and one patient (3.8%) received chemotherapy and palliative resection for gastric cancer followed by lung metastasis. The most frequently used anti-PD-1 antibody was sintilimab, followed by nivolumab, camrelizumab, and toripalimab in different patients.

**Table 2:** First treatment patterns of HER2-positive AFPGC

Treatment pattern	Number	%
Chemotherapy + Trastuzumab + anti-PD-1	16	61.5
SOX + Trastuzumab + anti-PD-1	13	50.0
XELOX + Trastuzumab + anti-PD-1	3	11.5
Chemotherapy + Trastuzumab	4	15.4
SOX + Trastuzumab	2	7.7
PF + Trastuzumab	1	3.8
FLOT + Trastuzumab	1	3.8
Chemotherapy + anti-PD-1	3	11.5
SOX + anti-PD-1	2	7.7
FLOT + anti-PD-1	1	3.8
Chemotherapy	2	7.7
SOX	1	3.8
XELOX	1	3.8
Paclitaxel	1	3.8
Chemotherapy + palliative resection	1	3.8
SOX + palliative resection	1	3.8

The most common chemotherapies used in this analysis were SOX (S-1 and oxaliplatin) and XELOX (capecitabine and oxaliplatin) (69.2% and 15.4%, respectively). The median number of treatment cycles was 5.96 cycles. In addition, two patients (7.7%) received FLOT, one patient (3.8%) received paclitaxel-based treatment, and one patient (3.8%) received FP. The details of the treatments are shown in Table 2.

For response evaluation, two patients did not have evaluation results available. In the remaining 24 patients, the ORR was 66.7%, and the DCR was 91.7%. Among the 16 patients in the quadruple combination group, 12 patients achieved a partial response, and the ORR was 80.0%. Stable disease (SD) was observed in 2 patients, and the DCR was 93.3%. The AFP level was greater than 1000 ng/ml, and the D-dimer level was greater than 1000 ng/ml in the 2 patients with rapid disease progression. The treatment results are shown in Table 3.

**Table 3:** Response evaluation of HER2-positive AFPGC patients receiving different treatments

Assessment	Chemotherapy + Trastuzumab + anti-PD-1 (*N* = 16)	Chemotherapy + Trastuzumab (*N* = 3)	Chemotherapy + anti-PD-1 (*N* = 3)	Chemotherapy (*N* = 2)	Chemotherapy + palliative resection (*N* = 1)
	**No**	**No**	**No**	**No**	
CR	0	0	0	0	
PR	12	3	1	0	
SD	2	0	1	2	1
PD	2	0	0	0	
unknown	1	0	1	0	
ORR	80.0%	100%	50%	0	0
DCR	93.3%	100%	100%	100%	100%

### Survival and associated factors in HER2-positive AFPGC patients

**Fig. 3:**
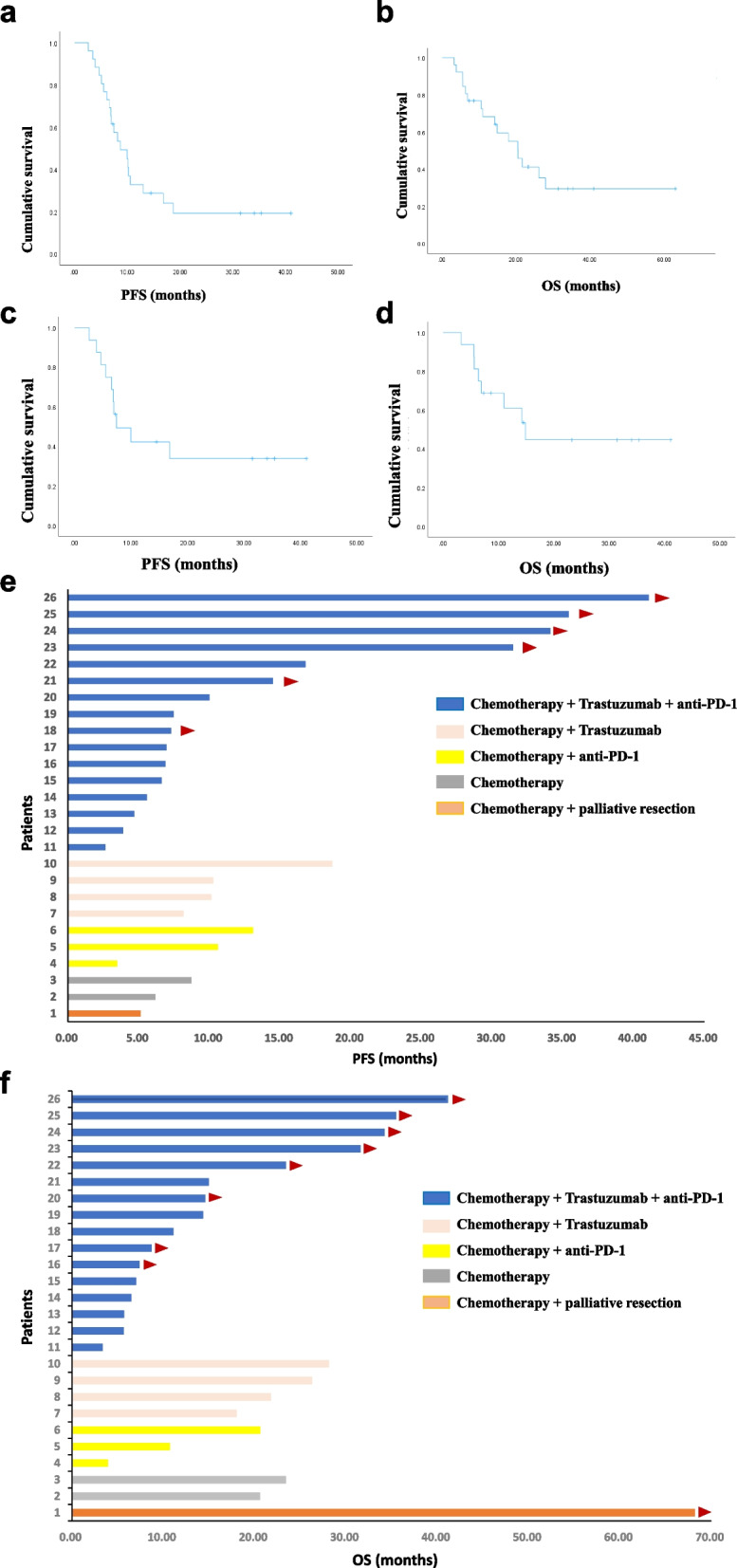
Survival curves were generated via the Kaplan–Meier method. **A** PFS of all patients; (**B**) OS of all patients; (**C**) PFS of patients receiving chemotherapy + trastuzumab + anti-PD-1 antibody; (**D**) OS of patients receiving chemotherapy + trastuzumab + anti-PD-1 antibody; (**E**) PFS of patients receiving different treatments; (**F**) OS of patients receiving different treatments. Two patients with an OS of less than one month and without treatments were not included in the PFS and OS analyses

By the cutoff date, 20 patients had disease progression after first-line treatment, and 16 patients had died. The median progression-free survival (mPFS) and overall survival (mOS) for all patients were 8.73 months (range: 5.57–11.89 months, Fig. 3A) and 20.47 months (range: 12.39–28.56 months, Fig. 3B), respectively. The median PFS and OS were 7.47 m and 14.87 months, respectively, in the patients in the chemotherapy + trastuzumab + anti-PD-1 antibody group, and there were still 6 patients without disease progression and 8 patients alive (PFS and OS range: 7.3–41.07 months; Fig. 3C and D). In the quadruplet group, 4 patients had PFS longer than 30 months. All patients who received chemotherapy + trastuzumab, chemotherapy + anti-PD-1 antibody therapy and chemotherapy alone died except one patient who underwent palliative resection for gastric cancer and lung metastasis. The PFS and OS rates of patients receiving different treatments are shown in detail in Fig. 3E and F.

**Fig.4:**
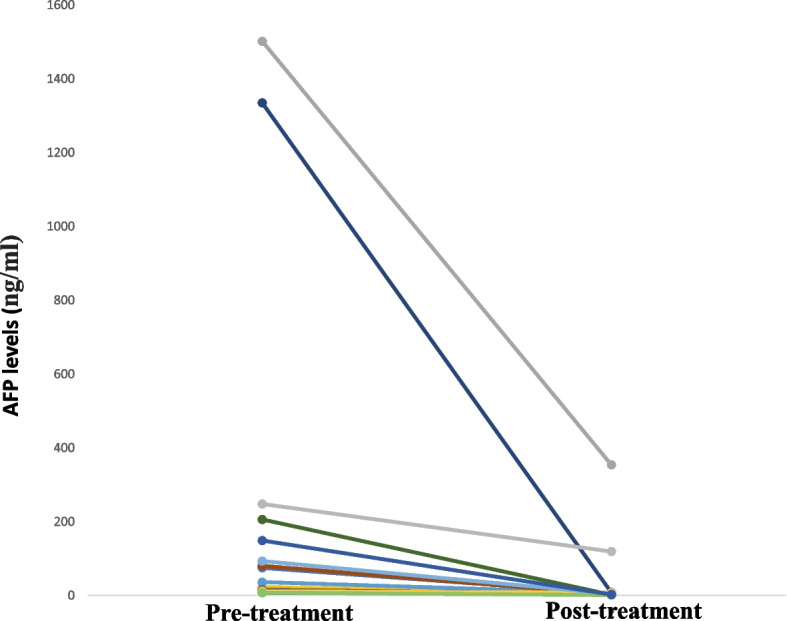
changes of AFP after treatments in patients

To further investigate the possible factors associated with the clinical outcomes of HER2 + AFPGC patients, the Kaplan‒Meier method was used for univariate analysis. As shown in Table 4, clinical parameters, AFP levels, pathological characteristics, metastatic information, and several possible associated biochemical markers from previous publications were included in the analysis. Based on the results of the univariate analysis, the serum AFP level, number of metastatic organs and degree of peritoneal metastasis were further included in the multivariate Cox proportional hazards regression model. However, we did not observe any correlations between AFP levels and clinical survival. The small sample size and anticancer therapy may have contributed to these results (Table 5). Most importantly, AFP levels decreased in all patients after treatment. The percentage of AFP decrease ranged from 31 to 99% (Fig. 4). More metastatic sites and peritoneal metastasis were associated with worse clinical outcomes (Supplemental Figs. 1 and 2). The details of the patients, treatments and survival rates are listed in Supplemental Table 1, with information such as specific adverse events or thrombosis clearly affecting the survival of the patients.

**Table 4:** Cox univariate analysis of AFP + HER2 + GC patients receiving first-line treatment

**Factor**	**PFS**				**OS**		
	***P***	**HR**	**95%CI**	***P***		**HR**	**95%CI**
*Clinical parameters*							
Gender (Male vs. Female)	0.917	0.978	0.641–1.491	0.789		1.095	0.562–2.137
Age (≥ 60 vs. < 60)	0.33	0.673	0.304–1.493	0.674		1.299	0.384–4.394
Smoking (yes vs. no)	0.795	0.901	0.412–1.974	0.862		1.108	0.348–3.526
Drinking (yes vs. no)	0.373	1.438	0.647–3.194	0.431		1.636	0.48–5.579
*AFP level at diagnosis*							
10–100 vs. $\le$ 10	0.389	1.783	0.479–6.647	0.261		0.673	0.338–1.342
100–1000 vs. $\le$ 10	0.862	1.105	0.357–3.418	0.677		1.158	0.581–2.309
≥ 1000 vs. $\le$ 10	0.803	1.248	0.220–7.096	0.748		0.939	0.640–1.377
*Pathologic differentiation*							
Moderate/Poor-Moderate vs. Poor	0.524	0.784	0.370–1.658	0.549		0.785	0.356–1.733
Metastatic organs at diagnosis							
Number of metastatic organs							
2 vs. 1	0.312	1.611	0.639–4.064	0.244		2.556	0.527–12.394
≥ 3 vs. 1	0.006	5.35	1.617–17.703	0.026		9.271	1.31–65.604
Metastatic sites							
Liver metastasis (yes vs. no)	0.582	1.255	0.560–2.812	0.988		0.991	0.301–3.266
Lymph node (yes vs. no)	0.229	0.182	0.011–2.923	0.441		26.773	0.006–114471.995
Peritoneal (yes vs. no)	0.015	3.139	1.252–7.867	0.063		3.181	0.937–10.798
*Biochemical markers*							
CEA level (< 20 vs. ≥ 20)	0.473	0.855	0.558–1.311	0.463		0.776	0.395–1.527
D-dimer level (< 2000 vs. ≥ 2000)	0.142	0.745	0.502–1.104	0.132		0.641	0.359–1.144
LDH (< 300 vs. ≥ 300)	0.71	0.9	0.519–1.563	0.383		0.702	0.317–1.554
ALB level (< 40 vs. ≥ 40)	0.803	1.118	0.466–2.68	0.538		0.618	0.133–2.862

*HR: hazard ratio, CI: confidence interval, PFS: progression-free survival, OS: overall survival*

**Table 5:** K‒M univariate analysis of AFP + HER2 + GC patients receiving first-line treatments

Factor	PFS			OS		
	***P***	**HR**	**95%CI**	***P***	**HR**	**95%CI**
Number of metastatic organs	0.373			0.135		
AFP level at diagnosis	0.789			0.518		
Peritoneal metastasis (yes vs. no)	0.002	0.371	0.198–0.695	0.009	0.444	0.241–0.820

*HR: hazard ratio, CI: confidence interval, PFS: progression-free survival, OS: overall survival*

### Immune microenvironment in HER2-positive AFPGC

**Fig. 5:**
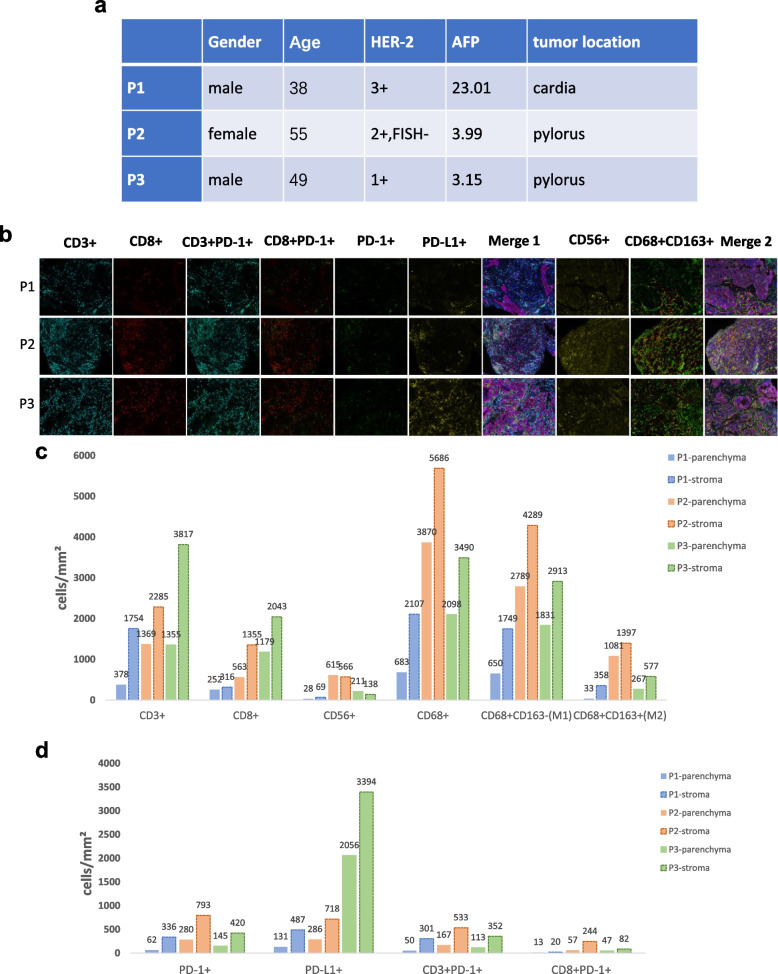
**A** Patient characteristics with multiple immunofluorescence stains; (**B**) images of immune cells with multiple immunofluorescence stains; (**C**) numbers of immune cells in the tumor parenchyma and stroma in three patients; (**D**) numbers of PD-1 + and PD-L1 + cells in the tumor parenchyma and stroma in three patients

We analyzed the immune microenvironment of three patients with advanced gastric cancer (Fig. 5A), one with elevated AFP and HER-2 3 + (Patient 1, P1) and the other two with normal AFP and HER-2 negativity (P2 and P3). An image of multiple immunofluorescence staining is shown in Fig. 5B. CD3 +, CD8 +, CD56 +, CD68 +, CD68 + CD163-(M1) and CD68 + CD163 + (M2) immune cells in the tumor parenchyma and stroma were significantly fewer in P1 than in P2 and P3 (Fig. 5C), as were PD-1 +, PD-L1 +, CD3 + PD-1 + and CD8 + PD-1 + immune cells (Fig. 5D).

The ratios of immune cells in the tumor parenchyma to those in the stroma were subsequently calculated. The ratios of CD3 +, CD56 +, CD68 +, CD68 + CD163- (M1) and CD68 + CD163 + (M2) immune cells in the tumor parenchyma to those in the stroma were obviously low at P1; most obviously, the number of CD56 + immune cells in the tumor parenchyma at P2 and P3 was greater than that in the stoma. The low numbers and ratios of immune cells in the tumor parenchyma in P1 suggested a local immunosuppressive microenvironment in AFP-elevated HER-2-positive gastric cancer. Interestingly, the ratios of CD8 + immune cells in the tumor parenchyma to those in the stroma were obviously high (P1, 79.7%; P2, 41.5%; and P3, 57.7%) (Supplemental Table 2).

The ratios of M2/M1, CD3PD-1 + and CD8 + PD-1 + immune cells were calculated. The M2/M1 ratio in the tumor parenchyma of P1 was 5.1%, which was the lowest among the three patients (P2 38.8% and P3 14.6%). The percentage of CD3PD-1 + immune cells in tumor parenchyma was 80.6%, which was the highest of the three patients (Supplemental Table 3). These findings suggest that patients with HER-2-positive AFPGC might benefit from treatment with anti-PD-1 antibodies, trastuzumab and chemotherapy.

## Discussion

First reported by Bourreill et al*.* [28] in 1970, gastric cancer with elevated serum AFP levels and liver metastasis was reported. With increasing research, concentrated interest has increased because of its aggressive behavior and poor prognosis. However, owing to its currently low incidence and ambiguous definition, our knowledge of this rare type of GC is currently far from adequate. Recent conclusions are mainly based on several retrospective studies with limited sample sizes. Li et al*.* analyzed 11 clinical studies on the Chinese AFPGC population and summarized the clinicopathological features as poor pathological characteristics, deep infiltration, increased numbers of lymph nodes and liver metastases, and shorter survival [29]. Although considered a rare subtype of GC, no specific clinical recommendations are currently available. With the success of the ToGA trial and the publication of interim results from the KEYNOTE-811 trial, targeted therapy and combined immunotherapy have shown great potential in HER2-positive GC patients. These findings raise the question of whether HER2-positive AFPGC patients can still benefit from the survival advantages of the current treatments for HER2-positive GC. Therefore, in this study, we focused on HER2-positive AFPGC patients, a population that has received little attention in previously published studies, and attempted to provide the first data on the clinicopathological features and clinical outcomes of HER2-positive AFPGC patients.

In this retrospective analysis, out of 1833 patients, 259 patients had an abnormally high AFP level greater than 7 ng/ml. The proportion of AFPGC reached 14.1%, which is higher than the 6–8% reported in previous studies. The lower cutoff level and late-stage enrollment may explain this result. A total of 33 patients (12.7%) were HER2 positive (2 +/ISH + or 3 +), and 4 patients were excluded from the final analysis because they were ultimately confirmed to have early-stage disease. The HER2 positivity rate was 12.7%, which was lower than that reported in some studies because some patients with HER2 2 + status were not confirmed by FISH. More than 70% of the selected patients presented with more than one metastatic site at initial diagnosis, with more than half having liver metastasis, which is consistent with the findings of a previous publication [30]. In addition, approximately one-third of patients have peritoneal metastasis, which is recognized as a poor prognostic biomarker [31, 32]. Additionally, two patients were excluded from the survival analysis because of a lack of systematic treatments due to poor physical status, and both died within one month, which may indicate the poor clinical characteristics and aggressive behavior of AFPGC. Immune microenvironment analysis with multiple immunofluorescence stains revealed immune inhibition in HER2-positive AFPGC compared with that in HER2-negative and AFP-normal gastric cancer. This is the first paper that describes the survival of AFPGC patients with HER-2 positive and presents the details of each patient.

Owing to the confounding effects of the treatments used in this analysis, we cannot draw any valid conclusions about the advantages and disadvantages of different treatment patterns. Among the 26 patients who received systematic anticancer treatment, more than half received a quadruple combination of anti-PD-1 immunotherapy, anti-HER2 targeted therapy and chemotherapy. With a median follow-up of 10 February 2023, the median PFS and median OS for all patients were 10.27 months and 20.5 months, respectively. In addition, 4 patients demonstrated PFS of more than 2 years without disease progression at the last follow-up. This is in parallel with the first interim analysis of the phase III KEYNOTE-811 study, in which the addition of anti-PD-1 therapy to trastuzumab and chemotherapy for unresectable or metastatic HER2-positive gastric or gastroesophageal junction adenocarcinoma yielded encouraging results, with an ORR of 74.4% and a DCR of 96.2% [22]. There was significant heterogeneity for the survival of the four-drug treatment group, which is not only related to high rates of liver and peritoneal metastasis (58.6% and 34.5%), but also to the occurrence of arterial and venous thrombosis in some patients. The prognosis of patients with thrombosis was very poor. Moreover, four patients who received trastuzumab plus chemotherapy achieved a median PFS of 10.13 months and an OS of 21.7 months, which is not inferior to the results of the phase III ToGA trial [16]. Although high serum AFP levels indicate poor prognosis in patients with gastric cancer, HER2-positive AFPGC patients can still receive equivalent survival benefits from the current treatment pattern for patients with HER2-positive gastric cancer. Immune microenvironment analysis revealed fewer immune cells in the tumor parenchyma and stroma in HER-2-positive AFPGC patients. This finding suggested the immune suppression of HER-2-positive AFPGC. The high ratio of CD8 + and CD3 + PD-1 + cells might be related to the effect of immunotherapy.

Previously published data revealed that several biological biomarkers, including D-dimer [33], carcinoembryonic antigen (CEA) and ALB levels, may be associated with survival in patients with AFPGC. Therefore, in this study, basic clinical parameters such as age, sex, personal history, treatment patterns together with pathological parameters and certain possible associated serum biomarkers were included in univariate analysis to detect the possible risk factors for AFPGC. In univariate analysis, patients with more than 2 metastatic sites, peritoneal metastasis and higher serum AFP levels were associated with shorter PFS and OS. In subsequent multivariate analysis, peritoneal metastasis, a feature previously reported by several studies to distinguish advanced disease and significant morbidity [31], was found to be an independent prognostic biomarker for the AFPGC. In our study, D-dimer and CEA were not obviously related to the survivals of the patients, because the number was not very big for HER-2 positive AFPGC patients. Our research has indeed revealed the presence of both venous and arterial thrombosis in four patients with D-dimer elevation. These patients all had very poor prognosis with OS ranging from 3.83 months to 6.90 months. D-dimer elevation may relate to high VEGF expression and abnormal blood clotting mechanism activation. This abnormal clotting mechanism has also led to the addition of anti-angiogenic drugs in current clinical trials [33–35].

Our work has several limitations, mainly due to its retrospective design. First, the retrospective observational study design may cause selection bias because the serum AFP level is not routinely detected in all gastric cancer patients, and only patients whose initial AFP level is available were included in this study; therefore, the proportion of patients included in this study may not fully represent the characteristics of the whole population. Second, even though this is the largest number of HER-2-positive AFPGC cases ever reported, the sample size in this study was not large enough because the proportion was small. Lastly, the quality of life, postchemotherapy cognitive impairment and language functions should be paid attention to [36–39]. Taken together, a subsequent randomized control study with a large sample size is warranted to further specify the treatment for HER2-positive AFPGC patients.

In conclusion, although AFPGC is now well accepted as a subgroup of GC patients with advanced disease and poor prognosis, some HER2-positive AFPGC patients may still derive equivalent survival benefits from current treatment methods focused on HER2 targeting. Peritoneal metastasis was an independent factor for shorter PFS. In addition, we speculated that in patients with good physical status, more potent quadruple combination therapy may have considerable clinical promise.

## Supplementary Information

Supplementary Material 1. Figure S1 PFS in different patients with different subgroup. Figure S2 OS in different patients with different subgroup.

Supplementary Material 2. Table S1 Details of the characteristics, treatments and survival of the patients. Table S2 Ratios of cells in the tumor parenchyma to those in the stroma. Table S3 Ratios of cells in the tumor parenchyma to those in the stroma.

## Data Availability

Data is provided within the manuscript and supplementary information files.
