# Supplementary material for: The combination of anti-PD-1 antibodies, trastuzumab and chemotherapy may improve the outcome of some patients with HER2-positive alpha-fetoprotein-producing gastric cancer: a retrospective real-world analysis from a single center
Source: BMC Cancer. 2025 Oct 10;25:1549. doi: 10.1186/s12885-025-14808-3 (PMC12512704; doi:10.1186/s12885-025-14808-3)
Supplement: Supplementary file 1 — Supplementary Material 1. Figure S1 PFS in different patients with different subgroup. Figure S2 OS in different patients with different subgroup. [file 12885_2025_14808_MOESM1_ESM.pdf]

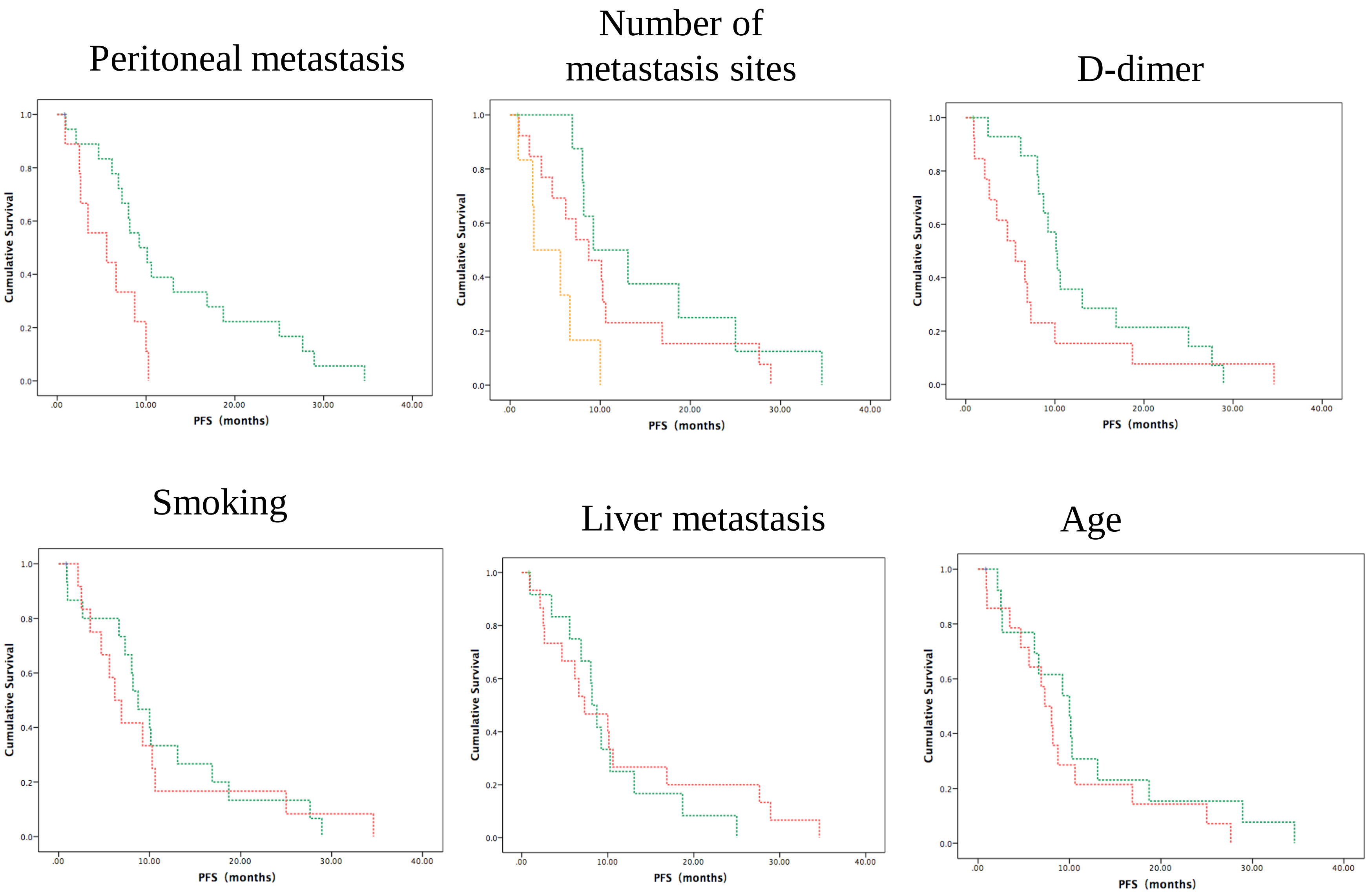

Figure S1 PFS in different patients with different subgroup

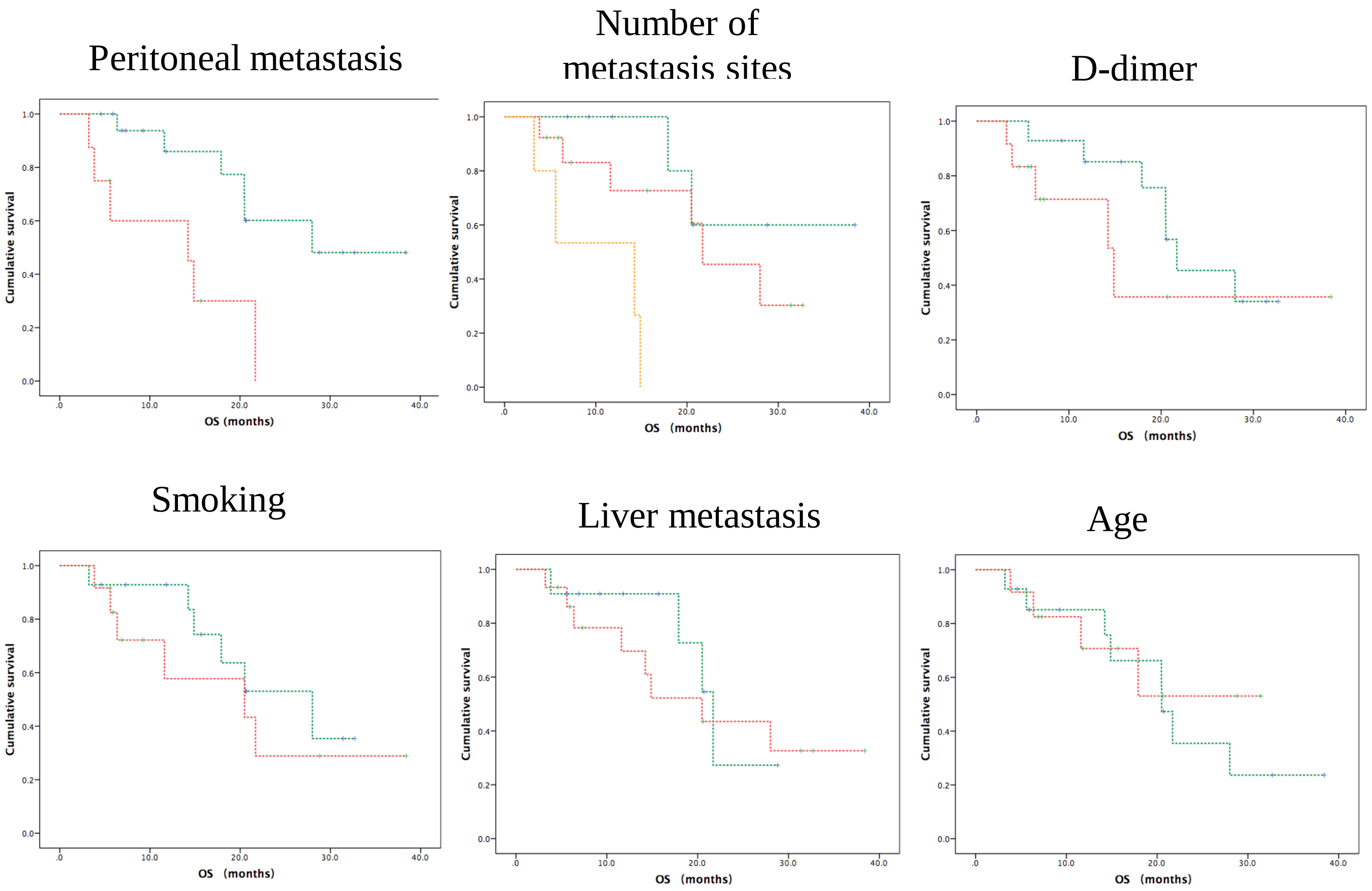

Figure S2 OS in different patients with different subgroup
