# Supplementary material for: The combination of anti-PD-1 antibodies, trastuzumab and chemotherapy may improve the outcome of some patients with HER2-positive alpha-fetoprotein-producing gastric cancer: a retrospective real-world analysis from a single center
Source: BMC Cancer. 2025 Oct 10;25:1549. doi: 10.1186/s12885-025-14808-3 (PMC12512704; doi:10.1186/s12885-025-14808-3)
Supplement: Supplementary file 2 — Supplementary Material 2. Table S1 Details of the characteristics, treatments and survival of the patients. Table S2 Ratios of cells in the tumor parenchyma to those in the stroma. Table S3 Ratios of cells in the tumor parenchyma to those in the stroma. [file 12885_2025_14808_MOESM2_ESM.docx]

**Supplemental Table 1 Details of the characteristics, treatments and survival of the patients.**

| patient No | gender | age | ECOG  score | tumor location | metastatic organs | AFP level  (ng/ml) | D-dimer  (ng/ml) | HER-2 status | treatment regimens | response evaluation | PFS for first line (months) | disease progression | OS（months） | Alive | Special Instructions |
| --- | --- | --- | --- | --- | --- | --- | --- | --- | --- | --- | --- | --- | --- | --- | --- |
| 1 | male | 58 | 1 | pylorus | lymph nodes | 9.63 | 343.5 | 2, FISH+ | SOX + palliative resection | SD | 33.40 | Yes | 68.10 | Yes |  |
| 2 | male | 52 | 1 | gastric body | liver and lymph nodes | 71.63 | 304.23 | 3 | XELOX | SD | 6.17 | Yes | 20.47 | No |  |
| 3 | female | 74 | 1 | gastric body | lymph nodes and peritoneum | 9.14 | 1789.74 | 3 | Paclitaxel | SD | 8.73 | Yes | 23.30 | Yes |  |
| 4 | male | 69 | 1 | gastric body, pylorus | peritoneum | 88.3 | 9991.14 | 3 | SOX + anti-PD-1 antibody | unknown | 3.47 | Yes | 3.83 | No | discontinue with Trastuzumab due to alergy and Venous thrombosis after the second cycle of treatment |
| 5 | male | 78 | 1 | cardia | liver and lymph nodes | 12 | 1854.3 | 2, FISH+ | SOX | SD | 10.60 | Yes | 10.60 | No |  |
| 6 | female | 51 | 1 | cardia | lymph nodes | 74.9 | 252.35 | 3 | FLOT + anti-PD-1 antibody | PR | 13.07 | Yes | 20.50 | No |  |
| 7 | male | 74 | 1 | cardia | lymph nodes | 15.5 | 1320.13 | 3 | SOX + Trastuzumab | PR | 8.17 | Yes | 17.90 | No |  |
| 8 | male | 58 | 1 | pylorus | liver and lymph nodes | 19.5 | 679.03 | 3 | FP+ Trastuzumab | unknown | 10.13 | Yes | 28.00 | No |  |
| 9 | male | 49 | 1 | pylorus | lymph nodes and peritoneum | 1092 | 1206.76 | 3 | SOX + Trastuzumab | PR | 10.27 | Yes | 21.70 | No |  |
| 10 | female | 48 | 1 | gastric body | lymph nodes | 8.92 | 2587.5 | 2, FISH+ | FLOT+ Trastuzumab | PR | 18.70 | Yes | 26.17 | No |  |
| 11 | female | 53 | 1 | gastric body, pylorus | liver, lymph nodes and peritoneum | 1210 | 3263.51 | 3 | SOX + Trastuzumab + anti-PD-1 antibody | PD | 2.63 | Yes | 3.23 | No |  |
| 12 | male | 51 | 1 | pylorus | liver, lymph nodes and peritoneum | 189243 | 1363.39 | 3 | SOX + Trastuzumab + anti-PD-1 antibody | PD | 3.90 | Yes | 5.60 | No | Venous thrombosis in left lower limb before treatment |
| 13 | male | 68 | 1 | pylorus | liver and lymph nodes | 1502 | 6581.16 | 3 | SOX + Trastuzumab + anti-PD-1 antibody | SD | 4.67 | Yes | 6.37 | No |  |
| 14 | male | 73 | 1 | pylorus | lymph nodes and peritoneum | 248 | >10000 | 3 | SOX + Trastuzumab + anti-PD-1 antibody | PR | 5.57 | Yes | 5.57 | No | Venous thrombosis in both lower limbs before treatment |
| 15 | female | 55 | 1 | Gastric body | liver, lymph nodes and peritoneum | 10.1 | 4172.82 | 3 | XELOX + Trastuzumab + anti-PD-1 antibody | PR | 6.63 | Yes | 14.87 | No |  |
| 16 | male | 60 | 1 | Whole stomach | lymph node | 7.84 | 4710.86 | 3 | SOX + Trastuzumab + anti-PD-1 antibody | PR | 6.90 | Yes | 6.90 | No | Arterial thrombosis in right lower limb after two-month treatment and then took S-1 till PD without other treatments later |
| 17 | male | 69 | 1 | pylorus | liver and lymph nodes | 93.3 | 6172.11 | 3 | SOX + Trastuzumab + anti-PD-1 antibody | PR | 6.97 | Yes | 11.00 | No | Interruption of anti-PD-1 treatment after 3 months due to immune enteritis |
| 18 | male | 45 | 1 | gastric body, pylorus | liver and lymph nodes | 149 | 4331.76 | 3 | SOX + Trastuzumab + anti-PD-1 antibody | PR | 7.30 | No | 7.30 | Yes |  |
| 19 | male | 51 | 1 | cardia | liver and lymph nodes | 7.49 | 7564.34 | 3 | SOX + Trastuzumab + anti-PD-1 antibody | PR | 7.47 | Yes | 8.60 | Yes | refused venous drugs after cycles treatment then took S-1 for one cycle before PD in first-line treatment |
| 20 | male | 42 | 0 | gastric body | liver, lymph nodes and peritoneum | 36.3 | 3699.02 | 3 | SOX + Trastuzumab + anti-PD-1 antibody | SD | 10.00 | Yes | 14.23 | No |  |
| 21 | female | 64 | 1 | cardia | lymph nodes | 10.9 | 1201.54 | 3 | SOX + Trastuzumab + anti-PD-1 antibody | PR | 14.50 | No | 14.50 | Yes |  |
| 22 | male | 61 | 1 | pylorus | liver and lymph nodes | 206 | 366.48 | 2, FISH+ | SOX + Trastuzumab + anti-PD-1 antibody | PR | 16.80 | Yes | 23.30 | Yes |  |
| 23 | male | 69 | 1 | cardia | lymph nodes and peritoneum | 80.4 | 1211.52 | 2, FISH+ | XELOX + Trastuzumab + anti-PD-1 antibody | PR | 31.47 | No | 31.47 | Yes |  |
| 24 | female | 67 | 1 | cardia | liver and lymph nodes | 13.4 | 1108.94 | 2, FISH+ | SOX + Trastuzumab + anti-PD-1 antibody | PR | 34.10 | No | 34.10 | Yes |  |
| 25 | male | 38 | 1 | cardia | liver and lymph nodes | 23.01 | 976.38 | 3 | SOX + Trastuzumab + anti-PD-1 antibody | PR | 35.40 | No | 35.40 | Yes |  |
| 26 | male | 54 | 1 | pylorus | liver | 1335 | 3847.72 | 3 | XELOX + Trastuzumab + anti-PD-1 antibody | PR | 41.07 | No | 41.07 | Yes |  |

**Supplemental Table 2 Ratios of cells in the tumor parenchyma to those in the stroma**

| Patients | CD3+ | CD8+ | CD56+ | CD68+ | CD68+CD163- | CD68+CD163+ | PD1+ | PD-L1 |
| --- | --- | --- | --- | --- | --- | --- | --- | --- |
| P1 | 21.6% | 79.7% | 40.6% | 32.4% | 37.2% | 9.2% | 18.5% | 26.9% |
| P2 | 59.9% | 41.5% | 108.7% | 68.1% | 65.0% | 77.4% | 35.3% | 39.8% |
| P3 | 35.5% | 57.7% | 152.9% | 60.1% | 62.9% | 46.3% | 34.5% | 60.6% |

**Supplemental Table 3 Ratios of cells in the tumor parenchyma to those in the stroma**

|  | M2/M1 | | CD3+PD-1+/PD-1+ | | CD8+PD-1+/PD-1+ | |
| --- | --- | --- | --- | --- | --- | --- |
|  | **parenchyma** | **stroma** | **parenchyma** | **stroma** | **parenchyma** | **stroma** |
| P1 | 5.1% | 20.5% | 80.6% | 89.6% | 21.0% | 6.0% |
| P2 | 38.8% | 32.6% | 59.6% | 67.2% | 20.4% | 30.8% |
| P3 | 14.6% | 19.8% | 77.9% | 83.8% | 32.4% | 19.5% |
